# Identification of Haplotype Tag Single-Nucleotide Polymorphisms within the PPAR Family Genes and Their Clinical Relevance in Patients with Major Trauma

**DOI:** 10.3390/ijerph13040374

**Published:** 2016-03-26

**Authors:** Jun-Wei Gao, Ling Zeng, An-Qiang Zhang, Xiao Wang, Wei Pan, Ding-Yuan Du, Lian-Yang Zhang, Wei Gu, Jian-Xin Jiang

**Affiliations:** 1 State Key Laboratory of Trauma, Burns and Combined Injury, Institute of Surgery Research, Daping Hospital, Third Military Medical University, Daping, Chongqing 400042, China; nutdgjw@163.com (J.W.G.); zengling_1025@126.com (L.Z.); zhanganqiang@126.com (A.-Q.Z.); kingb@tmmu.edu.cn (X.W.); qiangwei2@126.com (W.P.); hpzhangly@163.com (L.-Y.Z.); clgwjm@163.com (W.G.); 2 Chongqing Emergency Medical Center, Chongqing 40042, China; dingyuandu@sina.com

**Keywords:** peroxisome proliferator-activated receptor, genetic polymorphism, trauma, sepsis, MODS

## Abstract

Background: Peroxisome proliferator-activated receptors (PPARs) play important roles in the development of inflammatory diseases and sepsis. Recently, genetic variants of *PPARs* genes have been widely studied in some inflammatory diseases. However, the association between *PPAR* family of genes polymorphisms and sepsis risk in trauma patients was little known. Methods: SNPs were selected from the *PPARs* genes through constructing haplotype blocks and genotyped by the improved multiplex ligation detection reaction (iMLDR) method. The association between the selected SNPs and the risk of sepsis and multiple organ dysfunction (MOD) scores was evaluated in 734 trauma patients. In addition, tumor necrosis factor α (TNFα) production of peripheral blood leukocytes was also analyzed after lipopolysaccharide (LPS) stimulation. Results: Our results revealed that there were significant associations between the rs10865710 polymorphism and the risk of sepsis and MOD scores in Chinese Han trauma patients. Further, we found that the level of TNFα production was higher in patients with the rs10865710 G allele compared to those with the variant C allele. Conclusions: The rs10865710 polymorphism in the *PPAR*γ gene might be used to assess the risk of sepsis and multiple organ dysfunction syndrome (MODS) in trauma patients.

## 1. Introduction

Despite advances in the development of the clinical care system, trauma is still a major public health problem around the world, and its complications remain the main cause of in-hospital death in trauma patients. Sepsis and multiple organ dysfunction syndrome (MODS) are known as common and severe complications in trauma patients [1]. Thus, it important to prevent the development of sepsis and MODS in the management of trauma.

Peroxisome proliferator-activated receptors (PPARs) are transcription factors belonging to the nuclear receptor superfamily. There are three primary isoforms in the PPAR family: α, β/δ and γ, which are encoded by separate genes: *PPARA* (GenBank: NC_000022), *PPARD* (GenBank: NC_000006) and *PPARG* (GenBank: NC_000003) [2,3]. They form heterodimers with retinoid X receptors (RXRs) and bind to specific PPAR response elements (PPRE) in the promoter regions of target genes to regulate the transcription of a wide variety of genes [4]. We have known that the activation of PPARs plays a variety of important roles in energy metabolism, cell proliferation and differentiation, as well as inflammation [5,6,7,8]. Accumulating studies have focused on unraveling the association between PPARs and inflammatory diseases, such as inflammatory bowel disease, tumors and proteinuric kidney disease [9,10,11]. Their potential anti-inflammatory properties may be exerted most probably through inhibiting the NF-κB pathway [12,13,14], which is a typical signaling pathway in acute and chronic inflammatory diseases [15]. Under these circumstances, it is interesting to survey the potential roles of PPARs in the development of sepsis and MODS.

Recently, increasing studies have shown that single-nucleotide polymorphism (SNP) was an important genetic factor, which determined different inflammatory responses and clinical outcomes in trauma patients [16,17,18]. In addition, growing evidence has investigated the effect of allelic variation in *PPAR* family genes on inflammatory diseases, and this potential role may be achieved by influencing the magnitude of inflammatory response [19,20]. Therefore, it would be an important way to investigate the roles of PPARs in the development of sepsis and MODS by elucidating the clinical association between SNPs in *PPARs* genes and patients’ different responses to trauma. Although a previous study has assessed the association between the Pro12Ala polymorphism of *PPAR*γ and sepsis mortality [21], little was known about the clinical relevance of other SNPs in PPAR family genes in relation to the development of sepsis and MODS in trauma patients.

In our study, we selected nine common SNPs identified in the Chinese Han Beijing (CHB) population within and around the three *PPAR* family genes to evaluate the clinical association between these SNPs and the risk of sepsis and MODS.

## 2. Materials and Methods

### 2.1. Study Population

There were 734 unrelated trauma patients in our study. All of them were Chinese Han population and lived in the Chongqing district, southwest China. Eligible trauma patients were consecutively admitted to the Department of Trauma Surgery in the Daping Hospital and the Chongqing Emergency Medical Center from 1 January 2005–1 January 2014. The entry criteria were as follows: (1) patients’ age between 18 and 65 years; (2) patients with combined injury and their expected Injury Severity Score (ISS) >16; (3) patients surviving more than 48 h. Patients were excluded if they had penetrating injuries or preexisting cardiovascular, respiratory, renal, hepatic, hematologic or immunologic diseases. The ISS of each patient was calculated according to the abbreviated injury scale by two independent investigators in order to reduce personal error [22]. Standard surgical care and postoperative treatment were conducted among postoperative patients in the intensive care unit. Standard demographic, laboratory and clinical data of included trauma patients were extracted from a prospectively-collected database. Our study was approved by the Institutional Ethics Review Board (IERB) of Third Military Medical University (IERB No. TMMU2012009). Informed consent was obtained from the patients or their next of kin. The confidentiality of patients was preserved based on the guidelines for studies of human subjects.

### 2.2. Clinical Evaluation

Trauma patients were monitored prospectively after admission by physicians who did not know their genotypes. The diagnosis of sepsis was according to the criteria recommended by the American College of Chest Physicians and Society of Critical Care Medicine Consensus Conference [23]. Pneumonia was diagnosed when a predominant organism was isolated from appropriately-obtained sputum cultures in the setting of purulent sputum production and/or a new or changing pulmonary infiltrate on chest film. Bloodstream infections were identified according to the isolation of the predominant organism from blood cultures obtained under sterile conditions. The diagnostic criteria of urinary tract infections were as followed: >10 white blood cells/high power field on microscopic examination or isolation of >10^5^ organisms/mL urine or >10^4^ organisms and the presence of symptoms. Catheter-related infections were diagnosed if infection was suspected and more than 15 colony forming units were isolated from catheter tips cultured. The diagnosis of wound infection was based on drainage of purulent material from the wound. The physiological and laboratory data of each day were obtained during hospital stay, and clinical events were recorded until hospital discharge or death. We calculated MOD scores by summing all individual organ scores on each hospital day simultaneously [24]. Neurological scoring was not evaluated, because all included patients were sedated. All scores and diagnoses were determined by individuals who did not know the patients’ genotypes.

### 2.3. SNP Selection

In our study, the observed sequence of the human PPARα, β/δ and γ genes contained 3 kb upstream of the transcription start site, all exons, all introns and 3 kb downstream of the stop codon (99.16 kb, 91.62 kb and 152.5 kb total, respectively), which were located in chromosome 22, position 46150547–46243756 (PPARα), chromosome 6, position 35342558–35428191 (PPARβ/δ) and chromosome 3, position 12287850–12471013 (PPARγ), respectively [25].

We obtained all genetic variation data of the observed sequence of the human *PPAR*α*,* β*/*δ and γ genes from the CHB population of the HapMap project (www.hapmap.org). There were 620 SNPs (167 in *PPAR*α, 172 in *PPAR*β*/*δ and 281 in *PPAR*γ) identified in this database (Table S1). Among them, 164 SNPs (26 in *PPAR*α, 40 in *PPAR*β*/*δ and 98 in *PPAR*γ) have a minor allele frequency (MAF) of more than or equal to 0.05 (Table S2). Haploview (Version 4.2; Broad Institute of MIT and Harvard, Cambridge, MA, USA), a software package that provides computation of LD statistics and population haplotype patterns from genotype data, was used to construct haplotype blocks throughout the entire *PPAR*α*,* β*/*δ and γ genes, respectively [26]. A haplotype block represents the region inherited without substantial recombination in the ancestors of the current population. The history of recombination between a pair of SNPs can be estimated by using the normalized measure of allelic association D′ (value of D prime between the two loci) [27]. The criterion for the selected SNPs to construct a haplotype block is as follows: all SNPs in one region must be in strong LD with D′ of greater than 0.98 for the upper 95% confidence bound and greater than 0.7 for the lower bound.

### 2.4. Genotyping of Selected SNPs

Blood specimens of trauma patients were collected immediately after admission to avoid the effect of blood transfusion. We extracted genomic DNA from whole blood using the Wizard genomic DNA purification kit (Promega, Madison, WI, USA) according to the manufacturer’s protocol. Ultraviolet spectrophotometry was used to determine DNA concentration in all samples. Then the DNA concentration of each sample was adjusted to a 40-μg/mL concentration with sterile distilled water, we stored it at −80 °C. The improved multiplex ligation detection reaction (iMLDR) technique was used for genotyping according to a previous report [28]. Genotyping was performed by the operators, who did not know the clinical data of trauma patients. To ensure the quality of our results, about 10% of the samples were genotyped in duplicate.

### 2.5. Ex Vivo Tumor Necrosis Factor α Production

A human whole-blood assay was used according to our previous method [17]. After trauma patients were admitted, aliquots of whole blood were collected immediately and mixed 1:1 with Roswell Park Memorial Institute (RPMI) 1640 culture medium (Thermo Scientific, Beijing, China). Then, samples were incubated with 100 ng/mL LPS (Escherichia coli O26: B6, Difco Laboratories, Detroit, MI, USA) in a sample mixer at 37 °C for 4 h. The supernatants were collected after centrifugation and stored at −80 °C. A sandwich enzyme linked immunosorbent assay was used to detect the production of TNFα in the supernatants according to the manufacturer’s instructions (Endogen, Woburn, MA, USA).

### 2.6. Statistical Analysis

Our sample size was evaluated by online Power and Sample Size Program software [29]. We set the desired power at 80% with a significance level of 0.05 in a two-sided test and chose the log-additive inheritance model, which is the most suitable for polygenic diseases.

Allele frequencies for each SNP were determined by gene counting. Hardy–Weinberg equilibrium (HWE) was tested using chi-square (χ^2^) analyses based on our samples’ genotypes. Haploview (Version 4.2) software was selected to determine the extent of pairwise linkage disequilibrium between SNPs. The association between SNPs and MOD scores was performed using analysis of age, sex ratio and injury severity to adjust for possible confounding effects. Three genetic models (the dominant, recessive and allele comparison models) were included in our study for the unknown genetic model of sepsis. The association between SNPs and sepsis morbidity rate was determined by χ^2^ analyses. Odds ratios (ORs) and their corresponding 95% confidence intervals (CIs) were calculated by multiple logistic regression analyses to estimate the risk of sepsis. An independent-samples *t*-test was used to assess statistical significance for the comparison of TNFα levels. When the *p*-value was <0.05 after multiple testing, we considered it statistically significant. All statistical analyses were performed by SPSS software (Version 18.0; SPSS Inc., Chicago, IL, USA).

## 3. Results

### 3.1. Construction of Haplotype Blocks and Selection of SNPs

One-hundred and sixty-four common SNPs with a MAF ≥ 0.05 were shown within or around the PPAR family genes in the Chinese Han Beijing (CHB) population. There were nine haplotype blocks constructed by Haploview software: four haplotype blocks in the *PPAR*α gene, two haplotype blocks in the *PPAR*β gene and three haplotype blocks in the *PPAR*γ gene. Data are shown in Figures S1–S3. Finally, there were nine SNPs (rs135551, rs5769178, rs4253711, rs4823613, rs6902123, rs2016520, rs4684846, rs10865710 and rs1822825) included in our study (Table 1). All of them were located in the intron, except for rs2016520 (5′UTR) and rs10865710 (Extron A2).

Genetic variation data for the *PPAR* family genes was obtained from the HapMap project for 137 members of the Chinese Han Beijing (CHB) population.

### 3.2. Allele Frequencies and Genotype Distribution of the Selected SNPs among Trauma Patients

In our study cohort, the MAF of nine selected SNPs is shown in Table 2. These results were quite similar to the data from the CHB population in the HapMap database. In addition, the genotype distribution of all nine selected SNPs was consistent with HWE. All of these results suggested the stability of both allele and genotype frequencies for our selected SNPs in our study population. Therefore, they are in equilibrium from generation to generation.

### 3.3. Overall Clinical Characteristics of Patients with Major Trauma

There were 734 Chinese Han trauma patients in the Chongqing district in our study. The characteristics of our included patients are described in Table 3. All patients survived at least 48 h after admission. Among them, three hundred patients have developed sepsis in our population cohort, and pathogens were found in the blood cultures of 138 septic patients (46.0%). Respiratory tract infection was the main type of infection in our study cohort. The common pathogens identified in our study were *Staphylococcus aureus*, coagulase-negative staphylococci, *Klebsiella pneumoniae*, *Acinetobacter baumannii*, *Pseudomonas aeruginosa*, *Escherichia coli*, Enterococcus sp. and Enterobacter cloacae. Gram-negative infections made up about 21.7%; Gram-positive infections accounted for 11.3%; and mixed infections comprised about 9.7%, respectively. Organ dysfunction was found in 374 (51.0%) individuals in our study among whom, 116 (31.0%) had two or more organ dysfunctions. Among the patients with MODS, those with sepsis accounted for 77.6%.

### 3.4. Clinical Relevance of the Nine Selected SNPs with Development of Sepsis and MODS in Trauma Patients

In our study, no significant differences were found in age, gender ratio or ISS among patients with different genotypes of each SNP. There was a significant association between the rs10865710 polymorphism in the *PPAR*γ gene and the risk of sepsis and MODS in trauma patients. The patients carrying the G allele of the rs10865710 polymorphism significantly increased the risk of sepsis and MODS when compared to those carrying the C allele (*p* = 0.002 for the development of sepsis, *p* = 0.041 for MOD scores in the case of the recessive effect and *p* = 0.046 for the development of sepsis in the case of the dominant effect). Data from regression analyses after adjusting for age, sex ratio and ISS further indicated that the association of this polymorphism had a significant allele-dose effect with sepsis morbidity rate (OR = 1.461, 95% CI: 1.159–1.843, *p* = 0.001) (Table 4). However, other SNPs were not significantly associated with the risk of sepsis or MODS in our present study.

### 3.5. Effect of rs10865710 on LPS-Induced TNFα Production

**Figure 1:**
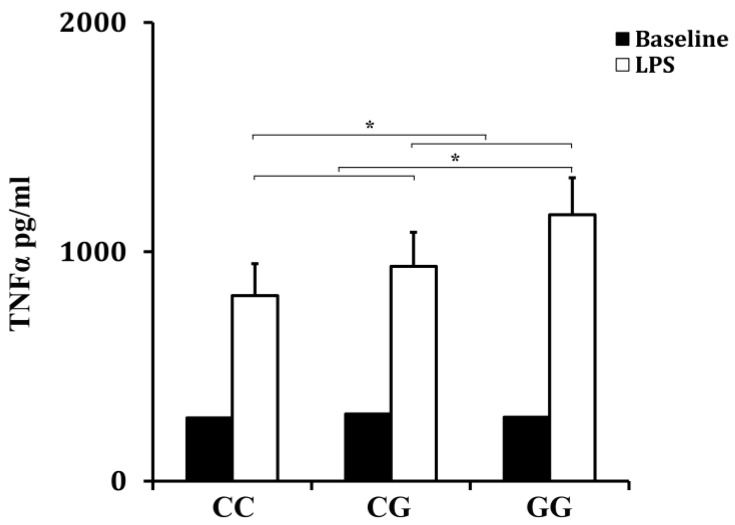
Effect of the PPARγ rs10865710 polymorphism on LPS-induced TNFα production (*p* = 0.041 for the dominant model; *p* = 0.027 for the recessive model).

The whole-blood samples were collected from trauma patients immediately after admission. Sixty patients were included in this part (*n* = 20 for the CC genotype, *n* = 20 for the GC genotype and *n* = 20 for the GG genotype). As shown in Figure 1, the rs10865710 polymorphism was associated with the LPS responsiveness of peripheral blood leukocytes. The level of TNFα production was higher in patients with the variant G allele compared to those with the variant C allele (*p* = 0.027 for the recessive effect and *p* = 0.041 for the dominant effect).

## 4. Discussion

It is commonly accepted that the inappropriate inflammatory response of the host organism to the invasion of microorganisms plays a fundamental role in the development of sepsis, and the generation of inflammatory factors could contribute to vascular coagulation and MODS. Despite the development of medicine, sepsis remains the leading cause of death in critically-ill patients. Therefore, identification of the high risk factors of sepsis and MODS seems important to develop effective therapies.

PPARs are ligand-activated nuclear receptors with a variety of effects on metabolism, cell differentiation, proliferation and apoptosis. Recent evidence has shown that PPARs were linked to inflammation and may play a key role in inflammation-related biological and pathological processes. PPARα and PPARγ are expressed in numerous tissues and inversely associated with inflammation-related factors, such as tumor necrosis factor α (TNFα), interleukin-1 beta (IL-1β) and other downstream markers of inflammation. This process may be achieved by either directly controlling the transcription process or indirectly inhibiting the activation of NF-κB [10,30], which plays a key role in the pathophysiology of sepsis. Compared to PPARα and γ, PPARβ/δ is ubiquitously expressed, but its biological role remains relatively unclear, and no PPARβ/δ drugs are used in the clinic. A previous study revealed that over-expression of PPARβ/δ could attenuate gene expression of TNFα, IL-1β and IL-6 in alveolar macrophages and played a protective role in sepsis-induced acute lung injury [31]. In addition, growing evidence from genetic association studies suggested that genetic factors may affect the inflammatory response and outcome of sepsis in trauma patients. Thus, we hypothesized that common genetic variants within PPARs may contribute to the development of sepsis and MODS in trauma patients.

In our study, we investigated the potential clinical relevance of the SNPs of *PPAR* family genes in trauma patients by means of the tag SNP of haplotype block. Nine SNPs (rs135551, rs5769178, rs4253711, rs4823613, rs6902123, rs2016520, rs4684846, rs10865710 and rs1822825) were identified within the whole *PPAR* family genes in the Chinese Han population. Our results showed that the rs10865710 in the *PPAR*γ gene revealed a strong clinical relevance with a higher rate of sepsis and MOD scores in the trauma patients with the variant G allele. Although the other SNPs have been reported to be associated with diabetes, myocardial infarction, obesity, hyperlipidemia and cancer in previous studies, they were not associated with the risk of sepsis or MODS in our present cohort.

Based on the functional significance of the rs10865710 polymorphism in the *PPAR*γ gene, we further investigated the association of this SNP with TNFα production from trauma patients to confirm our conclusion. Results showed that the capacity of peripheral leukocytes to produce TNFα was associated with the rs10865710 polymorphism in the *PPAR*γ gene: the patients who carried the variant G allele had a higher level of TNFα production, which was in agreement with its clinical relevance. Function analysis has shown that the rs10865710 polymorphism could alter the binding of the signal transducer and activator of transcription 5B (STAT5B) on the promoter of PPARγ, which may contribute to the reduced expression of PPARγ [32]. Further, PPARγ could affect the NF-κB signaling pathway and the downstream proinflammatory cytokines, which were seen as important factors during the development of sepsis and MODS. Taken together, the rs10865710 polymorphism might reduce the expression of PPARγ and then promote the activation of the NF-κB signaling pathway, which contributes to the increased TNFα production by peripheral blood leukocytes, leading to increased risk of sepsis and MODS in patients with major trauma.

The limitations of our study should be mentioned. Firstly, the number of trauma patients in our cohort was moderate, and all patients were limited to the Chongqing district. The clinical relevance of the rs10865710 polymorphism needs to be validated in a larger population. Secondly, we could not further investigate the PPARγ mRNAs levels in trauma patients due to the limitation of obtaining enough blood samples. Thirdly, our study population was restricted to Han Chinese, and we should further investigate the results in other ethnic groups. Fourthly, we did not detect the cell number of leukocytes in the blood of patients with different genotypes due to the limitation of samples. Finally, sepsis is a complex clinical syndrome related to systemic inflammation, and we could not take all factors into consideration due to the limited information.

## 5. Conclusions

In our study, the rs10865710 polymorphism in the *PPAR*γ gene could affect TNFα production and was associated with the development of sepsis and MODS in Chinese Han trauma patients. It might be used to estimate the risk of sepsis and MODS in trauma patients. Further studies based on larger sample sizes and more ethnicities are needed to confirm the significance of these findings.

## Figures and Tables

**Table 1:** SNPs identified within the PPAR family genes.

Gene	rs Number	Location	Variation	MAF ^1^ (%)	Region
*PPAR*α	rs135551	6523	G/A	8.4	Intron 2
	rs5769178	14776	A/C	15.7	Intron 2
	rs4253711	48535	G/A	15.6	Intron 3
	rs4823613	51809	A/G	21.7	Intron 3
*PPAR*β	rs6902123	20087	T/C	5.6	Intron 2
	rs2016520	68444	T/C	26.3	5′-UTR
*PPAR*γ	rs4684846	9501	G/A	46.0	Intron 1
	rs10865710	23850	C/G	34.9	Extron A2
	rs1822825	120615	G/A	45.3	Intron 5

^1^ Minor allele frequency.

**Table 2:** Distribution of the nine genotyped SNPs in trauma patients.

SNPs	Number	MAF ^1^ (%)		Genotypes, Number (%)			HWE ^3^ Test
		Patients	Database ^2^	Wild-Type	Heterozygous	Variant	
rs135551	734	7.6	8.4	628 (85.6)	101 (13.8)	5 (0.6)	0.67
rs5769178	734	15.0	15.7	525 (71.5)	198 (27.0)	11 (1.5)	0.11
rs4253711	734	14.4	15.6	543 (74.0)	170 (23.2)	21 (2.8)	0.09
rs4823613	734	23.2	21.7	436 (59.4)	256 (34.9)	42 (5.7)	0.59
rs6902123	734	3.1	5.6	690 (94.0)	42 (5.7)	2 (0.3)	0.12
rs2016520	734	30.4	26.3	357 (48.6)	308 (42.0)	69 (9.4)	0.83
rs4684846	727	45.8	46.0	208 (28.6)	372 (51.2)	147 (20.2)	0.41
rs10865710	734	34.6	34.9	316 (43.1)	328 (44.7)	90 (12.2)	0.73
rs1822825	732	45.5	45.3	215 (29.4)	368 (50.3)	149 (20.3)	0.71

^1^ Minor allele frequency; ^2^ data are derived from the HapMap database for Chinese Han in Beijing (*n* = 137); ^3^ Hardy–Weinberg equilibrium.

**Table 3:** Overall clinical characteristics of trauma patients.

Clinical Characteristics	Patient Data (*n* = 734)
Mean age ± SD, years	41.3 ± 12.1
Age range, years	18–65
Males/females, n	591/143
Mean ISS ^1^ ± SD	22.3 ± 9.4
≥16 to <25, n	435
≥25, n	299
Injured body regions, n	
Head	391
Thorax	473
Abdomen	280
Extremities	436
Number of regions injured, n	
Two	272
Three	188
All four	66
Organ dysfunction, n (%)	374 (51.0%)
One, n	258
Two, n	95
Three or above, n	21
Sepsis, n (%)	300 (40.9%)
Source of infection (%)	
Respiratory tract infection	46.4
Primary bloodstream infection	19.6
Urinary tract infection	14.9
Catheter-associated infection	9.5
Wound infection	7.2
Others ^2^	2.4
Pathogens (positive blood cultures), %	
Gram-negative	21.7
Gram-positive	11.3
Fungi	3.3
Mixed Gram-negative and Gram-positive	9.7
Negative blood cultures	54.0

^1^ Injury Severity Score; ^2^ other sites of infection included soft-tissue infection, bone infection and ear infection.

**Table 4:** Clinical relevance of the selected SNPs among trauma patients in Chongqing District.

SNPs	Genotype	Number	Age, Years	Sex, Male/Female, n	ISS ^1^	Sepsis, n	MOD ^2^ Score
rs135551	AA	5	40.8 ± 16.6	3/2	15.6 ± 3.8	2	4.0 ± 2.2
	AG	101	40.0 ± 11.7	87/14	21.3 ± 8.1	36	4.5 ± 2.2
	GG	628	41.4 ± 12.1	501/127	22.5 ± 9.6	262	4.9 ± 2.6
rs5769178	AA	525	40.9 ± 12.6	429/96	21.8 ± 9.3	209	4.8 ± 2.5
	AC	198	42.5 ± 10.6	156/42	23.3 ± 9.1	86	4.9 ± 2.6
	CC	11	34.9 ± 7.6	6/5	28.9 ± 15.8	5	5.4 ± 2.7
rs4253711	AA	21	40.1 ± 14.2	16/5	23.3 ± 12.9	7	4.5 ± 2.1
	AG	170	40.4 ± 11.5	132/38	22.3 ± 8.8	62	4.7 ± 2.5
	GG	543	41.6 ± 12.2	443/100	22.3 ± 9.5	231	4.9 ± 2.5
rs4823613	AA	436	41.5 ± 12.1	351/85	22.2 ± 9.6	186	5.0 ± 2.5
	AG	256	41.2 ± 12.0	207/49	22.4 ± 9.1	100	4.9 ± 2.5
	GG	42	39.1 ± 12.9	33/9	22.8 ± 9.5	14	5.1 ± 2.6
rs6902123	CC	2	41.2 ± 5.7	2/0	25.0 ± 4.2	1	5.5 ± 2.1
	CT	42	40.5 ± 12.0	31/11	21.1 ± 8.4	16	3.9 ± 1.8
	TT	690	41.3 ± 12.1	558/132	22.4 ± 9.5	283	4.9 ± 2.5
rs2016520	CC	69	41.7 ± 10.9	53/13	22.5 ± 8.9	25	4.6 ± 2.5
	CT	308	41.1 ± 12.0	243/65	22.3 ± 9.9	131	4.8 ± 2.4
	TT	357	41.3 ± 12.4	292/65	22.2 ± 9.1	144	5.0 ± 2.6
rs4684846	AA	147	41.0 ± 11.6	117/30	23.1 ± 9.1	63	5.3 ± 2.7
	AG	372	41.1 ± 12.4	302/70	22.0 ± 9.4	146	4.7 ± 2.3
	GG	208	41.8 ± 11.9	166/42	22.2 ± 9.8	89	5.0 ± 2.7
rs10865710	CC	316	41.0 ± 11.9	257/59	22.3 ± 10.0	116	4.9 ± 2.5
	CG	328	41.3 ± 12.3	263/65	22.3 ± 9.0	134	4.6 ± 2.3
	GG	90	41.9 ± 12.0	71/19	22.4 ± 9.0	50	5.8 ± 3.0
						a1, b1	a2
rs1822825	AA	149	41.9 ± 11.9	117/32	21.1 ± 9.3	60	4.9 ± 2.4
	AG	368	40.9 ± 12.2	299/69	22.6 ± 9.1	156	4.8 ± 2.6
	GG	215	41.4 ± 12.1	174/41	22.6 ± 10.1	83	4.9 ± 2.4

^1^ Injury Severity Score; ^2^ multiple organ dysfunction; a1, *p* = 0.002; a2, *p* = 0.041 for the recessive model; b1, *p* = 0.046 for the dominant model.

## Supplementary Materials

The following are available online at www.mdpi.com/1660-4601/13/4/374/s1, Figure S1: Linkage disequilibrium (LD) plot of the SNPs with a MAF ≥ 5% within the PPARα gene and 3 kb up- and down-stream regions are displayed using an r^2^ black and white color scheme. Black represents very high LD (r^2^ = 1), and white indicates the absence of correlation (r^2^ = 0) between SNPs. The selected SNPs and SNPs that are indirectly measured by them are listed with corresponding r^2^ values. The alleles of selected SNPs are given with their frequencies, according to the HapMap data for Chinese individuals from Beijing, Figure S2: Linkage disequilibrium (LD) plot of the SNPs with a MAF ≥ 5% within the PPARβ gene and 3 kb up- and down-stream regions are displayed using an r^2^ black and white color scheme. Black represents very high LD (r^2^ = 1), and white indicates the absence of correlation (r^2^ = 0) between SNPs. The selected SNPs and SNPs that are indirectly measured by them are listed with corresponding r^2^ values. The alleles of selected SNPs are given with their frequencies, according to the HapMap data for Chinese individuals from Beijing, Figure S3: Linkage disequilibrium (LD) plot of the SNPs with a MAF ≥ 5% within the PPARγ gene and 3 kb up- and down-stream regions are displayed using an r^2^ black and white color scheme. Black represents very high LD (r^2^ = 1), and white indicates the absence of correlation (r^2^ = 0) between SNPs. The selected SNPs and SNPs that are indirectly measured by them are listed with corresponding r^2^ values. The alleles of selected SNPs are given with their frequencies, according to the HapMap data for Chinese individuals from Beijing, Table S1: The total SNPs identified from the 137 healthy Chinese Han Beijing (CHB) individuals of the HapMap project, Table S2: The total common SNPs with a MAF ≥ 5% identified from the 137 healthy Chinese Han Beijing (CHB) individuals of the HapMap project.

## Abbreviations

The following abbreviations are used in this manuscript:

PPAR	peroxisome proliferator-activated receptor
iMLDR	improved multiplex ligation detection reaction
MOD	multiple organ dysfunction
TNFα	tumor necrosis factor α
LPS	lipopolysaccharides
MODS	multiple organ dysfunction syndrome
RXR	retinoid X receptor
PPRE	PPAR response element
SNP	single-nucleotide polymorphism
CHB	Chinese Han Beijing
ISS	Injury Severity Score
HWE	Hardy–Weinberg equilibrium
OR	odds ratio
IL-1β	interleukin-1 beta
