# Supplementary material for: Identification of Haplotype Tag Single-Nucleotide Polymorphisms within the PPAR Family Genes and Their Clinical Relevance in Patients with Major Trauma
Source: Int J Environ Res Public Health. 2016 Mar 26;13(4):374. doi: 10.3390/ijerph13040374 (PMC4847036; doi:10.3390/ijerph13040374)
Supplement: Supplementary file 1 [file ijerph-13-00374-s001.pdf]

Identification of Haplotype Tag Single Nucleotide Polymorphisms within the PPAR Family Genes and Their Clinical Relevance in Patients with Major Trauma

Jun-Wei Gao, Ling Zeng, An-Qiang Zhang, Xiao Wang, Wei Pan, Ding-Yuan Du, Lian-Yang Zhang, Wei Gu and Jian-Xin Jiang

Table S1. The total SNPs identified from the 137 healthy Chinese Han Beijing (CHB) individuals of the HapMap project.

Gene	No.	Name	Position	MAF	Alleles
PPARα	1	rs9615487	44923635	0	A/G
	2	rs4253781	44926043	0	C/T
	3	rs4253617	44927264	0	A/G
	4	rs4253621	44928018	0	A/G
	5	rs4253622	44928055	0	C/G
	6	rs4253623	44928770	0.174	A/G
	7	rs4253630	44930682	0	A/G
	8	rs135552	44931479	0.067	C/T
	9	rs6008970	44931664	0.011	C/T
	10	rs135551	44931685	0.081	A/G
	11	rs5769129	44931719	0	C/T
	12	rs6007965	44931786	0	A/G
	13	rs135550	44931898	0.078	C/T
	14	rs16994915	44931919	0	A/C
	15	rs135549	44931972	0.25	C/T
	16	rs135548	44932167	0.067	A/G
	17	rs135547	44932314	0.078	C/G
	18	rs8139731	44933016	0	C/T
	19	rs2238833	44933264	0.012	A/G
	20	rs4253783	44933960	0	A/G
	21	rs135543	44933985	0.07	C/T
	22	rs5769142	44934006	0	A/G
	23	rs4253633	44934334	0	A/G
	24	rs4253634	44934435	0	G/T
	25	rs4253635	44934650	0	A/G
	26	rs135542	44934701	0.067	C/T
	27	rs4253784	44934799	0	A/G
	28	rs6008974	44935586	0	A/G
	29	rs129600	44935825	0.337	A/G
	30	rs12160659	44936352	0.011	G/T
	31	rs16994933	44937241	0	C/T
	32	rs9306498	44937555	0	A/G
	33	rs6008976	44937677	0	A/G
	34	rs9680676	44938138	0	C/G
	35	rs9615523	44939431	0	C/T
	36	rs12166902	44939924	0	C/T
	37	rs5769178	44939938	0.221	A/C
	38	rs9626729	44940216	0	A/G

Table S1. *Cont.*

Gene	No.	Name	Position	MAF	Alleles
	39	rs9627046	44940230	0.209	A/G
	40	rs11913649	44940741	0	A/G
	41	rs4253640	44942002	0	C/G
	42	rs135538	44943292	0.43	C/G
	43	rs5769184	44944441	0	A/C
	44	rs881740	44946052	0.209	A/G
	45	rs5769219	44947547	0	G/T
	46	rs4253653	44947566	0	G/T
	47	rs4253655	44947835	0	A/G
	48	rs4253786	44948413	0	A/T
	49	rs9626736	44948896	0.151	A/G
	50	rs9626737	44949006	0	A/G
	51	rs12160049	44949046	0	A/C
	52	rs6009058	44949617	0	C/T
	53	rs4253662	44951408	0	A/G
	54	rs4253788	44952315	0	C/T
	55	rs4253678	44952674	0	A/G
	56	rs4253679	44952679	0.012	A/G
	57	rs6009081	44955815	0	A/G
	58	rs1555208	44955874	0	G/T
	59	rs11704856	44956144	0	A/T
	60	rs4253681	44958264	0.222	C/T
	61	rs4253682	44958361	0	G/T
	62	rs7289836	44958675	0	A/C
	63	rs4253684	44958811	0	C/T
	64	rs4253692	44959755	0	A/G
	65	rs9627100	44961405	0.012	A/G
	66	rs7287085	44961638	0	A/T
	67	rs761543	44963053	0	C/T
	68	rs7364220	44963648	0.012	A/G
	69	rs7364140	44963684	0	A/G
	70	rs4253698	44964092	0	C/T
	71	rs4253700	44964658	0	C/T
	72	rs4253792	44964718	0	C/T
	73	rs4253701	44964774	0.012	A/G
	74	rs4253702	44964950	0	A/G
	75	rs4253703	44965084	0	C/T
	76	rs4253704	44965370	0	C/G
	77	rs9626756	44965754	0	A/G
	78	rs11912612	44966079	0	G/T
	79	rs5769364	44966121	0	A/G
	80	rs5769366	44966162	0	A/G
	81	rs17661656	44966942	0	C/G
	82	rs12330015	44968942	0.209	A/G
	83	rs8138102	44970416	0.012	A/G
	84	rs11703495	44972000	0	A/T
	85	rs12170381	44972203	0	A/C
	86	rs4253711	44973697	0.156	A/G

Table S1. *Cont.*

Gene	No.	Name	Position	MAF	Alleles
	87	rs4253712	44973699	0.156	A/G
	88	rs7292459	44974766	0	C/T
	89	rs11913971	44974800	0	G/T
	90	rs7290473	44975951	0	A/C
	91	rs5766714	44975994	0	A/C
	92	rs4823613	44976971	0.221	A/G
	93	rs8139794	44977611	0	C/T
	94	rs12166868	44980224	0	G/T
	95	rs4253713	44980575	0	A/G
	96	rs4253719	44981311	0	A/G
	97	rs5766741	44983854	0.222	C/T
	98	rs5766743	44986042	0.163	A/G
	99	rs6008129	44988387	0	G/T
	100	rs4253727	44988626	0.011	C/T
	101	rs4253728	44988731	0	A/G
	102	rs4253729	44988897	0	C/G
	103	rs5767686	44989852	0	C/T
	104	rs4253736	44989925	0	C/G
	105	rs4253739	44990414	0	A/G
	106	rs4253743	44990971	0	A/G
	107	rs5767700	44991336	0.222	C/T
	108	rs4253744	44991568	0	C/G
	109	rs13056831	44991745	0	A/G
	110	rs4253747	44991901	0.198	A/T
	111	rs4253748	44991930	0	C/T
	112	rs6413511	44992811	0.022	A/G
	113	rs1800204	44992834	0.012	A/G
	114	rs1800206	44992938	0	C/G
	115	rs4253754	44993808	0	A/G
	116	rs4253755	44994040	0	A/G
	117	rs1800235	44994569	0	C/T
	118	rs4253756	44994722	0	C/T
	119	rs4253757	44994781	0	A/C
	120	rs12170204	44997864	0	C/T
	121	rs6008193	44998802	0	G/T
	122	rs6008197	44999080	0	C/G
	123	rs6007662	44999709	0.14	A/G
	124	rs5767743	45000658	0.256	C/T
	125	rs4253759	45000792	0	A/G
	126	rs4253760	45001048	0	G/T
	127	rs4253763	45001264	0	C/T
	128	rs4253764	45001452	0	A/C
	129	rs4253765	45001552	0	C/T
	130	rs6519979	45002201	0	C/T
	131	rs11090819	45005170	0	A/G
	132	rs4253772	45006267	0	C/T
	133	rs1042311	45006444	0	C/T
	134	rs4253798	45006948	0	A/G
	135	rs4253776	45008143	0	A/G

Table S1. *Cont.*

Gene	No.	Name	Position	MAF	Alleles
	136	rs4253777	45009286	0	A/G
	137	rs4253778	45009298	0	C/G
	138	rs4253779	45009457	0	C/T
	139	rs4253800	45010115	0	A/C
	140	rs5767779	45010515	0	A/T
	141	rs9615264	45011253	0	A/G
	142	rs11704979	45011658	0	A/G
	143	rs6008259	45012446	0.012	A/G
	144	rs11703765	45013924	0	C/T
	145	rs11703766	45013935	0	C/T
	146	rs4823487	45013985	0	A/T
	147	rs12167302	45014144	0	C/T
	148	rs3892755	45014263	0	A/G
	149	rs9615784	45014671	0.012	A/G
	150	rs7286168	45015134	0	C/T
	151	rs5766804	45015639	0	C/G
	152	rs10427717	45015701	0	C/T
	153	rs9626814	45015918	0	A/G
	154	rs4592961	45016034	0	C/T
	155	rs9627268	45016310	0	C/T
	156	rs10154348	45016823	0	A/C
	157	rs1055659	45016962	0	C/T
	158	rs11704508	45017204	0	A/G
	159	rs12159525	45017242	0	A/C
	160	rs14842	45018121	0	A/T
	161	rs6008306	45018756	0	A/G
	162	rs10154668	45018774	0	A/G
	163	rs5767842	45019491	0	C/T
	164	rs12159251	45019935	0	C/T
	165	rs7284616	45020136	0	C/T
	166	rs9626816	45020262	0	A/G
	167	rs11705226	45021238	0	A/C
PPARβ	1	rs9658056	35417538	0.067	C/T
	2	rs9658057	35417635	0	A/C
	3	rs9658059	35418444	0	C/T
	4	rs6919734	35419966	0	A/G
	5	rs2267664	35420232	0.233	A/G
	6	rs9462073	35420918	0	A/G
	7	rs9658068	35421954	0.093	A/G
	8	rs9658069	35422466	0.011	C/T
	9	rs9658070	35422846	0	A/C
	10	rs9658071	35423103	0.012	A/G
	11	rs9658072	35423118	0	A/G
	12	rs9658073	35423968	0	A/C
	13	rs9658074	35424109	0	C/T
	14	rs6457813	35425279	0.044	C/T
	15	rs4713851	35426762	0	A/G
	16	rs7771323	35428426	0.011	A/G
	17	rs6937510	35428789	0	C/T

Table S1. *Cont.*

Gene	No.	Name	Position	MAF	Alleles
	18	rs7744392	35430741	0.011	A/G
	19	rs7758272	35430953	0.012	A/G
	20	rs13206834	35431267	0	A/C
	21	rs9942489	35431687	0.011	A/T
	22	rs11758212	35431799	0	A/G
	23	rs9942465	35432191	0	A/C
	24	rs7746988	35432719	0.035	C/T
	25	rs4278009	35433696	0	C/T
	26	rs9658076	35434964	0	C/T
	27	rs9658077	35435339	0.012	A/G
	28	rs9658079	35435555	0	C/T
	29	rs9462075	35435794	0	C/T
	30	rs11967065	35436295	0	C/G
	31	rs12530232	35437384	0	G/T
	32	rs6901410	35438008	0.058	C/T
	33	rs11961212	35438128	0.012	C/T
	34	rs9348972	35438289	0.011	C/T
	35	rs6902123	35438399	0.056	C/T
	36	rs6457815	35438876	0.012	C/T
	37	rs11751895	35439082	0	C/T
	38	rs11962571	35440131	0	A/T
	39	rs9470001	35441719	0.057	C/G
	40	rs12181514	35441777	0	A/C
	41	rs7757196	35443268	0	A/G
	42	rs9658080	35445175	0.012	A/G
	43	rs9658081	35445249	0.012	C/T
	44	rs9658083	35445269	0.034	C/G
	45	rs9658084	35445334	0.058	C/T
	46	rs9296148	35445445	0.058	A/G
	47	rs9470003	35445909	0	C/G
	48	rs7754530	35445936	0	G/T
	49	rs7772754	35446084	0	C/G
	50	rs7739752	35447013	0.058	C/T
	51	rs9368862	35447527	0	C/T
	52	rs11751306	35447722	0	A/C
	53	rs9368863	35447899	0	C/T
	54	rs12173582	35448278	0.244	C/T
	55	rs6913026	35448872	0	C/G
	56	rs7750906	35448999	0.012	G/T
	57	rs12524787	35449114	0	G/T
	58	rs9470004	35449828	0	C/T
	59	rs6919334	35449853	0.058	A/G
	60	rs9658085	35452404	0.012	C/T
	61	rs9470007	35457717	0.058	C/T
	62	rs7770619	35458020	0.012	C/T
	63	rs6937483	35458737	0	A/G
	64	rs6922548	35461501	0.058	A/G
	65	rs9394294	35461533	0	A/G
	66	rs16868709	35462449	0	C/G

Table S1. *Cont.*

Gene	No.	Name	Position	MAF	Alleles
	67	rs12174594	35463391	0	C/T
	68	rs9348973	35463404	0.012	A/G
	69	rs9470011	35463423	0	C/T
	70	rs3777744	35464121	0.291	A/G
	71	rs9658098	35464509	0	A/G
	72	rs9658099	35464514	0	G/T
	73	rs9658100	35464618	0.058	G/T
	74	rs9658101	35464761	0	A/G
	75	rs3798343	35465671	0.233	C/G
	76	rs9658107	35465763	0	C/T
	77	rs9462078	35465952	0	A/G
	78	rs6915115	35466923	0.012	C/T
	79	rs6457816	35470826	0.058	C/T
	80	rs9658108	35471735	0.011	C/G
	81	rs9658112	35472771	0	C/T
	82	rs9658115	35473024	0	A/G
	83	rs9658117	35473389	0.023	G/T
	84	rs2284197	35473435	0.044	A/G
	85	rs9658118	35473806	0	A/G
	86	rs9658119	35473945	0.012	A/C
	87	rs9380506	35474868	0.193	A/C
	88	rs1040436	35475887	0.256	A/G
	89	rs10081143	35476761	0	C/T
	90	rs9470015	35477062	0.189	A/G
	91	rs2267665	35477469	0.198	A/G
	92	rs1883322	35477784	0.256	C/T
	93	rs1883321	35477812	0	C/T
	94	rs2267666	35478706	0.256	A/T
	95	rs9658120	35479057	0	C/T
	96	rs9658121	35479278	0	A/G
	97	rs9658122	35479313	0	C/T
	98	rs9658123	35479323	0	A/G
	99	rs9658125	35479412	0.047	C/T
	100	rs9658126	35479533	0	C/T
	101	rs7751726	35479602	0.012	A/G
	102	rs7751481	35479731	0.256	A/G
	103	rs9658127	35480031	0	A/G
	104	rs9658128	35480060	0	C/T
	105	rs2267667	35480502	0.256	C/G
	106	rs9470017	35481971	0	A/C
	107	rs2038068	35482439	0.256	A/G
	108	rs2038067	35482444	0.198	A/G
	109	rs6906237	35483504	0.047	A/C
	110	rs9462081	35483984	0.012	A/C
	111	rs6911817	35484265	0	C/T
	112	rs4388282	35484328	0	A/C
	113	rs4555908	35484381	0	A/C
	114	rs4292520	35484392	0	A/C
	115	rs4587165	35484657	0	A/C

Table S1. *Cont.*

Gene	No.	Name	Position	MAF	Alleles
	116	rs9658129	35485692	0	A/G
	117	rs2267668	35485900	0.174	A/G
	118	rs9658130	35486018	0	A/G
	119	rs2267669	35486102	0.174	A/G
	120	rs9658131	35486713	0	G/T
	121	rs9658133	35486739	0	C/T
	122	rs2016520	35486756	0.233	C/T
	123	rs9658134	35486776	0	A/G
	124	rs9658135	35486975	0	A/G
	125	rs9658136	35487324	0	C/T
	126	rs9658137	35487533	0	A/G
	127	rs9658138	35487549	0	G/T
	128	rs9658144	35488111	0	A/G
	129	rs9296153	35488200	0	C/T
	130	rs9658145	35488292	0	C/T
	131	rs9658146	35488720	0	C/G
	132	rs6942084	35488968	0	A/G
	133	rs4713854	35489077	0	A/C
	134	rs9470019	35491163	0	A/G
	135	rs2299869	35491410	0.186	C/T
	136	rs9717879	35492207	0	G/T
	137	rs4513790	35492313	0	A/G
	138	rs12198825	35492451	0	A/G
	139	rs9462082	35494019	0.174	A/G
	140	rs9296154	35495356	0.047	A/G
	141	rs9658147	35495443	0	A/G
	142	rs9658148	35495458	0	A/G
	143	rs9658149	35495516	0	A/C
	144	rs7762809	35495718	0.011	C/T
	145	rs9658150	35495878	0	A/G
	146	rs9658151	35496362	0.012	C/T
	147	rs9658152	35496446	0	C/T
	148	rs2076169	35496457	0.174	A/G
	149	rs9658156	35497488	0	C/T
	150	rs9658158	35498077	0	A/G
	151	rs9658159	35499538	0	A/G
	152	rs2076167	35499765	0.233	C/T
	153	rs9658160	35499882	0	C/T
	154	rs9658161	35500005	0	C/G
	155	rs9658162	35500068	0	A/G
	156	rs9658163	35500245	0	C/T
	157	rs9658164	35500687	0	A/G
	158	rs2076166	35501382	0.186	A/G
	159	rs9658166	35501741	0	C/T
	160	rs9658168	35502225	0	A/T
	161	rs9470022	35502843	0	C/T
	162	rs9658175	35502880	0	C/T
	163	rs9658176	35502925	0	C/T
	164	rs3734253	35502939	0	A/C

Table S1. *Cont.*

Gene	No.	Name	Position	MAF	Alleles
	165	rs3734254	35502988	0.202	C/T
	166	rs9658177	35503426	0.105	A/G
	167	rs9658178	35503524	0	C/T
	168	rs1053046	35503556	0.035	A/G
	169	rs1053049	35503596	0.221	C/T
	170	rs760783	35503982	0.186	G/T
	171	rs7749160	35505105	0.186	C/T
	172	rs9470023	35506335	0.012	G/T
PPARγ	1	rs9837686	12301825	0	A/T
	2	rs13077495	12302751	0.023	C/T
	3	rs17029002	12303932	0	C/T
	4	rs2920502	12304195	0.244	C/G
	5	rs17036160	12304783	0.023	C/T
	6	rs17036183	12306536	0	C/T
	7	rs12106668	12308495	0	C/G
	8	rs2972164	12309416	0.116	C/T
	9	rs6809631	12310647	0.408	A/T
	10	rs6785890	12310816	0.43	A/C
	11	rs11709077	12311507	0.023	A/G
	12	rs6768587	12313115	0.43	A/G
	13	rs4684846	12313849	0.43	A/G
	14	rs9817428	12315267	0.43	A/C
	15	rs17036188	12315925	0.337	C/T
	16	rs12631028	12316406	0.462	C/T
	17	rs12636461	12316830	0.43	A/G
	18	rs13076055	12316996	0.43	A/G
	19	rs12631819	12317861	0.337	G/T
	20	rs11128596	12318117	0.43	A/C
	21	rs11710969	12318591	0.43	A/G
	22	rs7620165	12319441	0.058	A/G
	23	rs11128597	12319636	0.43	A/G
	24	rs17793693	12320971	0	A/C
	25	rs10510410	12321738	0.233	A/C
	26	rs10510411	12321849	0.233	A/G
	27	rs6805419	12321911	0	C/T
	28	rs10510412	12321962	0.222	A/G
	29	rs17036242	12324490	0.233	A/G
	30	rs13061415	12324924	0.233	C/T
	31	rs10212597	12325680	0	A/G
	32	rs12639162	12325781	0.233	A/G
	33	rs12485478	12326223	0.198	A/G
	34	rs12629240	12326574	0.233	A/G
	35	rs9310401	12327468	0.233	C/T
	36	rs11715073	12327971	0.233	C/G
	37	rs10865710	12328198	0.233	C/G
	38	rs3112394	12328303	0	C/T
	39	rs11128598	12328326	0.233	C/T
	40	rs13095716	12328612	0.222	A/T
	41	rs13073869	12328993	0.233	A/G

Table S1. *Cont.*

Gene	No.	Name	Position	MAF	Alleles
	42	rs12496505	12331158	0.222	A/G
	43	rs12487012	12331196	0.233	C/T
	44	rs13070963	12331605	0.233	C/T
	45	rs6800748	12332832	0	A/G
	46	rs6800910	12332957	0	A/G
	47	rs13433696	12333492	0.233	A/G
	48	rs2067819	12334049	0.023	A/G
	49	rs2067820	12334056	0	A/G
	50	rs12636454	12335214	0.233	C/T
	51	rs12633551	12335494	0.209	C/T
	52	rs11128599	12335769	0.233	A/G
	53	rs12495364	12336929	0.233	C/T
	54	rs7646425	12338291	0	A/G
	55	rs7646510	12338358	0	A/G
	56	rs12493718	12338637	0.233	G/T
	57	rs13083375	12340308	0.023	G/T
	58	rs3892175	12343038	0	A/G
	59	rs2920501	12343887	0	C/T
	60	rs13064760	12344401	0.023	C/T
	61	rs9814788	12344799	0.012	C/T
	62	rs880663	12345083	0.222	A/G
	63	rs17793951	12345737	0.058	A/G
	64	rs12496005	12346082	0.167	C/G
	65	rs4145574	12347074	0.233	A/G
	66	rs4145573	12347386	0.233	A/G
	67	rs2028760	12347882	0.233	A/G
	68	rs3112395	12348094	0	A/G
	69	rs1122648	12350391	0.233	C/T
	70	rs1984668	12350712	0	A/T
	71	rs9823137	12351178	0	C/G
	72	rs17036314	12351745	0.222	C/G
	73	rs10510417	12352294	0.222	C/G
	74	rs11128601	12356165	0.233	C/T
	75	rs9870196	12358664	0	A/G
	76	rs7638903	12358714	0.023	A/G
	77	rs12490265	12359542	0.133	A/G
	78	rs17036321	12359599	0	C/T
	79	rs12330440	12359870	0	C/G
	80	rs11128603	12360828	0.022	A/G
	81	rs4684847	12361337	0.023	C/T
	82	rs12635309	12362281	0	C/T
	83	rs10510418	12363563	0.116	A/C
	84	rs17036326	12364313	0.023	A/G
	85	rs12497191	12365135	0.209	A/G
	86	rs17036328	12365484	0.023	C/T
	87	rs11712510	12365695	0	C/T
	88	rs11712531	12365713	0	C/T
	89	rs6802898	12366207	0.023	C/T
	90	rs2197423	12366583	0.023	A/G

Table S1. *Cont.*

Gene	No.	Name	Position	MAF	Alleles
	91	rs7614873	12366809	0	G/T
	92	rs7647481	12366813	0.023	A/G
	93	rs17036333	12367181	0.035	A/G
	94	rs7649970	12367272	0.023	C/T
	95	rs4135301	12367518	0	A/C
	96	rs4135302	12367649	0	A/G
	97	rs4135245	12367792	0	C/G
	98	rs1801282	12368125	0.023	C/G
	99	rs4135303	12368317	0	C/T
	100	rs4135304	12369601	0	A/G
	101	rs1899951	12369840	0.024	C/T
	102	rs4135246	12370005	0	A/T
	103	rs4135305	12370233	0	A/G
	104	rs4684848	12370645	0.023	A/G
	105	rs7615916	12370958	0.233	A/G
	106	rs4135306	12371444	0	G/T
	107	rs4135247	12371588	0.369	A/G
	108	rs2881654	12371955	0.023	A/G
	109	rs17817276	12372392	0.14	A/G
	110	rs6788489	12372838	0	A/T
	111	rs6778740	12373636	0	C/T
	112	rs7627605	12374270	0	C/T
	113	rs12629751	12374407	0.209	C/T
	114	rs9851767	12374542	0	A/G
	115	rs9829551	12375963	0	A/G
	116	rs1064323	12377007	0	A/G
	117	rs1373641	12377474	0.128	C/T
	118	rs4135307	12377540	0	A/G
	119	rs4135308	12377582	0	C/T
	120	rs1373640	12377601	0.131	A/G
	121	rs9883281	12378731	0	A/G
	122	rs9812662	12379323	0	G/T
	123	rs4135253	12379999	0.14	-/T
	124	rs11718489	12383049	0	A/C
	125	rs4135311	12383089	0	A/T
	126	rs2938397	12383278	0.384	A/G
	127	rs4135313	12383519	0	A/G
	128	rs4135315	12383758	0	A/G
	129	rs4135316	12383821	0	C/T
	130	rs4135255	12383905	0	A/G
	131	rs4135317	12383950	0	G/T
	132	rs4135256	12384528	0	A/G
	133	rs17817469	12385349	0	C/T
	134	rs6801982	12385702	0	A/C
	135	rs9858822	12386238	0	A/C
	136	rs9842021	12386727	0	G/T
	137	rs2921188	12387115	0.14	A/G
	138	rs6442311	12387955	0	A/G
	139	rs964232	12388237	0	C/T

Table S1. *Cont.*

Gene	No.	Name	Position	MAF	Alleles
	140	rs2120825	12388339	0.023	G/T
	141	rs7650213	12388536	0	G/T
	142	rs7641290	12389325	0	C/T
	143	rs6809832	12389420	0	C/T
	144	rs13091131	12389478	0	A/C
	145	rs13091468	12389627	0	A/C
	146	rs4135320	12391182	0	A/G
	147	rs4135321	12391219	0	A/G
	148	rs4135259	12393604	0	C/T
	149	rs2028759	12393612	0.369	C/T
	150	rs4135322	12393759	0	C/T
	151	rs4135323	12394091	0	A/C
	152	rs2921190	12394475	0.349	C/T
	153	rs13306745	12396056	0.081	G/T
	154	rs11914306	12396492	0	A/C
	155	rs4135260	12397488	0	C/T
	156	rs4135261	12398038	0	C/T
	157	rs4135324	12398113	0	C/G
	158	rs4135263	12398266	0	C/T
	159	rs13316883	12399502	0	A/T
	160	rs2972162	12399793	0.349	C/T
	161	rs4135325	12400285	0	A/G
	162	rs10510419	12401936	0	G/T
	163	rs2959269	12404165	0	C/T
	164	rs2938395	12404468	0.36	A/G
	165	rs4135326	12404745	0	A/G
	166	rs4135264	12404810	0	A/G
	167	rs4135327	12404983	0	A/G
	168	rs2959268	12405075	0.349	C/T
	169	rs4135329	12405216	0	A/G
	170	rs4135266	12406550	0	A/T
	171	rs2938394	12406568	0.36	A/G
	172	rs2938393	12407068	0	A/C
	173	rs10510420	12408298	0	A/C
	174	rs4135333	12409091	0	A/C
	175	rs4135334	12409272	0	A/T
	176	rs2938392	12409608	0.36	A/G
	177	rs2938391	12409725	0	C/T
	178	rs4135335	12409733	0.012	C/T
	179	rs2938390	12409741	0	C/G
	180	rs2292101	12409901	0.244	C/T
	181	rs4371498	12411157	0	G/T
	182	rs4135268	12412237	0.056	C/G
	183	rs4135336	12412396	0	A/G
	184	rs4135337	12412533	0	C/G
	185	rs2921192	12413448	0	G/T
	186	rs4135340	12414314	0	A/G
	187	rs2938387	12414387	0.395	C/T
	188	rs9876829	12414687	0	A/T

Table S1. *Cont.*

Gene	No.	Name	Position	MAF	Alleles
	189	rs4135341	12416553	0	G/T
	190	rs4135342	12417578	0	G/T
	191	rs2959273	12417731	0.36	A/G
	192	rs2959272	12417833	0.36	G/T
	193	rs1875796	12418657	0.36	C/T
	194	rs4135275	12418844	0.477	A/G
	195	rs12489347	12418877	0.233	C/G
	196	rs9836965	12419275	0	C/G
	197	rs1151996	12420807	0.36	A/C
	198	rs4135276	12420830	0	C/T
	199	rs4135277	12420846	0	C/G
	200	rs1151997	12421004	0.36	A/G
	201	rs4135278	12421468	0	A/G
	202	rs11713869	12421481	0	G/T
	203	rs1151999	12422153	0.36	G/T
	204	rs1797895	12422814	0	C/G
	205	rs1152000	12422895	0	C/T
	206	rs1185785	12422950	0	C/T
	207	rs4135343	12422964	0.012	A/G
	208	rs4135344	12423142	0	A/G
	209	rs4135345	12423299	0	A/G
	210	rs4135280	12423994	0.244	C/T
	211	rs3127609	12424013	0	A/G
	212	rs4135347	12424026	0	C/T
	213	rs4135281	12424453	0	C/T
	214	rs796313	12424528	0.349	G/T
	215	rs4135282	12424559	0	C/T
	216	rs1373642	12424651	0	A/C
	217	rs796290	12424683	0.372	C/G
	218	rs4135283	12424793	0.244	A/C
	219	rs4135284	12424866	0	A/G
	220	rs1822825	12424963	0.36	A/G
	221	rs709149	12425354	0.36	A/G
	222	rs709150	12426337	0.36	C/G
	223	rs11128604	12426593	0	A/G
	224	rs9871668	12427388	0	A/T
	225	rs1987536	12429505	0	G/T
	226	rs709151	12429999	0.36	C/T
	227	rs4135348	12431456	0	G/T
	228	rs709154	12431834	0.375	A/T
	229	rs4135285	12432119	0	A/G
	230	rs4135350	12432324	0	A/G
	231	rs4135351	12432461	0	A/G
	232	rs4135352	12433208	0	C/T
	233	rs13306747	12433274	0.035	C/G
	234	rs13306746	12433334	0	C/T
	235	rs796289	12436368	0	C/T
	236	rs4135288	12436548	0	A/G
	237	rs709156	12436615	0.128	A/G

Table S1. *Cont.*

Gene	No.	Name	Position	MAF	Alleles
	238	rs4135354	12436640	0.012	C/T
	239	rs1699373	12436932	0	G/T
	240	rs709157	12437024	0.047	A/G
	241	rs7623946	12438717	0	A/G
	242	rs7645903	12438826	0	A/T
	243	rs7635807	12438970	0	C/T
	244	rs1175540	12440243	0.372	A/C
	245	rs4135292	12440956	0	A/G
	246	rs1175542	12441214	0.372	A/G
	247	rs1175543	12441433	0.384	A/G
	248	rs1177809	12441490	0.349	A/G
	249	rs4135293	12441596	0	C/T
	250	rs4135295	12441783	0	A/G
	251	rs4135296	12441939	0	C/T
	252	rs1175544	12442044	0.349	C/T
	253	rs2655259	12442543	0	C/T
	254	rs4135356	12442613	0	A/G
	255	rs7636598	12442827	0	A/G
	256	rs7636854	12443118	0	A/G
	257	rs7639159	12443273	0	A/G
	258	rs7616804	12443286	0	A/G
	259	rs7639005	12443299	0	C/G
	260	rs13099634	12443463	0	C/T
	261	rs13099828	12443589	0	C/G
	262	rs17036700	12443627	0	G/T
	263	rs3105364	12443690	0	A/G
	264	rs6442312	12444449	0.011	A/G
	265	rs6442313	12444667	0	A/G
	266	rs3773360	12444718	0	A/G
	267	rs4135359	12445043	0	A/G
	268	rs1797912	12445239	0.384	A/C
	269	rs4135360	12445314	0	A/G
	270	rs4135361	12445663	0	A/G
	271	rs6782475	12446330	0	G/T
	272	rs6794024	12446362	0	A/G
	273	rs4135362	12447095	0	A/G
	274	rs7626560	12450088	0	C/T
	275	rs7650895	12450162	0	A/C
	276	rs3856806	12450557	0.244	C/T
	277	rs1152003	12452055	0.477	C/G
	278	rs6790976	12452742	0	A/G
	279	rs9827787	12452816	0.013	A/G
	280	rs10222537	12453380	0	C/T
	281	rs9833097	12453817	0	A/G

Table S2. The total common SNPs with a MAF ≥ 5% identified from the 137 healthy Chinese Han Beijing (CHB) individuals of the HapMap project.

Gene	No.	rs Number	Position	MAF	Alleles	Location on the Gene	Region
PPARα	1	rs4253623	44928770	0.174	A/G	44928770	intron
	2	rs135552	44931479	0.067	C/T	44931479	intron
	3	rs135551	44931685	0.081	A/G	44931685	intron
	4	rs135550	44931898	0.078	C/T	44931898	intron
	5	rs135549	44931972	0.25	C/T	44931972	intron
	6	rs135548	44932167	0.067	A/G	44932167	intron
	7	rs135547	44932314	0.078	C/G	44932314	intron
	8	rs135543	44933985	0.07	C/T	44933985	intron
	9	rs135542	44934701	0.067	C/T	44934701	intron
	10	rs129600	44935825	0.337	A/G	44935825	intron
	11	rs5769178	44939938	0.221	A/C	44939938	intron
	12	rs9627046	44940230	0.209	A/G	44940230	intron
	13	rs135538	44943292	0.43	C/G	44943292	intron
	14	rs881740	44946052	0.209	A/G	44946052	intron
	15	rs9626736	44948896	0.151	A/G	44948896	intron
	16	rs4253681	44958264	0.222	C/T	44958264	intron
	17	rs12330015	44968942	0.209	A/G	44968942	intron
	18	rs4253711	44973697	0.156	A/G	44973697	intron
	19	rs4253712	44973699	0.156	A/G	44973699	intron
	20	rs4823613	44976971	0.221	A/G	44976971	intron
	21	rs5766741	44983854	0.222	C/T	44983854	intron
	22	rs5766743	44986042	0.163	A/G	44986042	intron
	23	rs5767700	44991336	0.222	C/T	44991336	intron
	24	rs4253747	44991901	0.198	A/T	44991901	intron
	25	rs6007662	44999709	0.14	A/G	44999709	intron
	26	rs5767743	45000658	0.256	C/T	45000658	intron
PPARβ	27	rs9658056	35417538	0.067	C/T	35417538	5′flanking
	28	rs2267664	35420232	0.233	A/G	35420232	intron
	29	rs9658068	35421954	0.093	A/G	35421954	intron
	30	rs6901410	35438008	0.058	C/T	35438008	intron
	31	rs6902123	35438399	0.056	C/T	35438399	intron
	32	rs9470001	35441719	0.057	C/G	35441719	intron
	33	rs9658084	35445334	0.058	C/T	35445334	intron
	34	rs9296148	35445445	0.058	A/G	35445445	intron
	35	rs7739752	35447013	0.058	C/T	35447013	intron
	36	rs12173582	35448278	0.244	C/T	35448278	intron
	37	rs6919334	35449853	0.058	A/G	35449853	intron
	38	rs9470007	35457717	0.058	C/T	35457717	intron
	39	rs6922548	35461501	0.058	A/G	35461501	intron
	40	rs3777744	35464121	0.291	A/G	35464121	intron
	41	rs9658100	35464618	0.058	G/T	35464618	intron
	42	rs3798343	35465671	0.233	C/G	35465671	intron
	43	rs6457816	35470826	0.058	C/T	35470826	intron
	44	rs9380506	35474868	0.193	A/C	35474868	intron
	45	rs1040436	35475887	0.256	A/G	35475887	intron
	46	rs9470015	35477062	0.189	A/G	35477062	intron
	47	rs2267665	35477469	0.198	A/G	35477469	intron

Table S2. *Cont.*

Gene	No.	rs Number	Position	MAF	Alleles	Location on the Gene	Region
	48	rs1883322	35477784	0.256	C/T	35477784	intron
	49	rs2267666	35478706	0.256	A/T	35478706	intron
	50	rs7751481	35479731	0.256	A/G	35479731	intron
	51	rs2267667	35480502	0.256	C/G	35480502	intron
	52	rs2038068	35482439	0.256	A/G	35482439	intron
	53	rs2038067	35482444	0.198	A/G	35482444	intron
	54	rs2267668	35485900	0.174	A/G	35485900	intron
	55	rs2267669	35486102	0.174	A/G	35486102	intron
	56	rs2016520	35486756	0.233	C/T	35486756	5′UTR
	57	rs2299869	35491410	0.186	C/T	35491410	intron
	58	rs9462082	35494019	0.174	A/G	35494019	intron
	59	rs2076169	35496457	0.174	A/G	35496457	intron
	60	rs2076167	35499765	0.233	C/T	35499765	synonymous codon
	61	rs2076166	35501382	0.186	A/G	35501382	intron
	62	rs3734254	35502988	0.202	C/T	35502988	3′UTR
	63	rs9658177	35503426	0.105	A/G	35503426	3′UTR
	64	rs1053049	35503596	0.221	C/T	35503596	3′UTR
	65	rs760783	35503982	0.186	G/T	35503982	3′flanking
	66	rs7749160	35505105	0.186	C/T	35505105	3′flanking
PPARγ	67	rs2920502	12304195	0.244	C/G	12304195	5′flanking
	68	rs2972164	12309416	0.116	C/T	12309416	intron
	69	rs6809631	12310647	0.408	A/T	12310647	intron
	70	rs6785890	12310816	0.43	A/C	12310816	intron
	71	rs6768587	12313115	0.43	A/G	12313115	intron
	72	rs4684846	12313849	0.43	A/G	12313849	intron
	73	rs9817428	12315267	0.43	A/C	12315267	intron
	74	rs17036188	12315925	0.337	C/T	12315925	intron
	75	rs12631028	12316406	0.462	C/T	12316406	intron
	76	rs12636461	12316830	0.43	A/G	12316830	intron
	77	rs13076055	12316996	0.43	A/G	12316996	intron
	78	rs12631819	12317861	0.337	G/T	12317861	intron
	79	rs11128596	12318117	0.43	A/C	12318117	intron
	80	rs11710969	12318591	0.43	A/G	12318591	intron
	81	rs7620165	12319441	0.058	A/G	12319441	intron
	82	rs11128597	12319636	0.43	A/G	12319636	intron
	83	rs10510410	12321738	0.233	A/C	12321738	intron
	84	rs10510411	12321849	0.233	A/G	12321849	intron
	85	rs10510412	12321962	0.222	A/G	12321962	intron
	86	rs17036242	12324490	0.233	A/G	12324490	intron
	87	rs13061415	12324924	0.233	C/T	12324924	intron
	88	rs12639162	12325781	0.233	A/G	12325781	intron
	89	rs12485478	12326223	0.198	A/G	12326223	intron
	90	rs12629240	12326574	0.233	A/G	12326574	intron
	91	rs9310401	12327468	0.233	C/T	12327468	intron
	92	rs11715073	12327971	0.233	C/G	12327971	intron
	93	rs10865710	12328198	0.233	C/G	12328198	extron A2
	94	rs11128598	12328326	0.233	C/T	12328326	intron
	95	rs13095716	12328612	0.222	A/T	12328612	intron

Table S2. *Cont.*

Gene	No.	rs Number	Position	MAF	Alleles	Location on the Gene	Region
	96	rs13073869	12328993	0.233	A/G	12328993	intron
	97	rs12496505	12331158	0.222	A/G	12331158	intron
	98	rs12487012	12331196	0.233	C/T	12331196	intron
	99	rs13070963	12331605	0.233	C/T	12331605	intron
	100	rs13433696	12333492	0.233	A/G	12333492	intron
	101	rs12636454	12335214	0.233	C/T	12335214	intron
	102	rs12633551	12335494	0.209	C/T	12335494	intron
	103	rs11128599	12335769	0.233	A/G	12335769	intron
	104	rs12495364	12336929	0.233	C/T	12336929	intron
	105	rs12493718	12338637	0.233	G/T	12338637	intron
	106	rs880663	12345083	0.222	A/G	12345083	intron
	107	rs17793951	12345737	0.058	A/G	12345737	intron
	108	rs12496005	12346082	0.167	C/G	12346082	intron
	109	rs4145574	12347074	0.233	A/G	12347074	intron
	110	rs4145573	12347386	0.233	A/G	12347386	intron
	111	rs2028760	12347882	0.233	A/G	12347882	intron
	112	rs1122648	12350391	0.233	C/T	12350391	intron
	113	rs17036314	12351745	0.222	C/G	12351745	intron
	114	rs10510417	12352294	0.222	C/G	12352294	intron
	115	rs11128601	12356165	0.233	C/T	12356165	intron
	116	rs12490265	12359542	0.133	A/G	12359542	intron
	117	rs10510418	12363563	0.116	A/C	12363563	intron
	118	rs12497191	12365135	0.209	A/G	12365135	intron
	119	rs7615916	12370958	0.233	A/G	12370958	intron
	120	rs4135247	12371588	0.369	A/G	12371588	intron
	121	rs17817276	12372392	0.14	A/G	12372392	intron
	122	rs12629751	12374407	0.209	C/T	12374407	intron
	123	rs1373641	12377474	0.128	C/T	12377474	intron
	124	rs1373640	12377601	0.131	A/G	12377601	intron
	125	rs4135253	12379999	0.14	-/T	12379999	intron
	126	rs2938397	12383278	0.384	A/G	12383278	intron
	127	rs2921188	12387115	0.14	A/G	12387115	intron
	128	rs2028759	12393612	0.369	C/T	12393612	intron
	129	rs2921190	12394475	0.349	C/T	12394475	intron
	130	rs13306745	12396056	0.081	G/T	12396056	intron
	131	rs2972162	12399793	0.349	C/T	12399793	intron
	132	rs2938395	12404468	0.36	A/G	12404468	intron
	133	rs2959268	12405075	0.349	C/T	12405075	intron
	134	rs2938394	12406568	0.36	A/G	12406568	intron
	135	rs2938392	12409608	0.36	A/G	12409608	intron
	136	rs2292101	12409901	0.244	C/T	12409901	intron
	137	rs4135268	12412237	0.056	C/G	12412237	intron
	138	rs2938387	12414387	0.395	C/T	12414387	intron
	139	rs2959273	12417731	0.36	A/G	12417731	intron
	140	rs2959272	12417833	0.36	G/T	12417833	intron
	141	rs1875796	12418657	0.36	C/T	12418657	intron
	142	rs4135275	12418844	0.477	A/G	12418844	intron
	143	rs12489347	12418877	0.233	C/G	12418877	intron

Table S2. *Cont.*

Gene	No.	rs Number	Position	MAF	Alleles	Location on the Gene	Region
	144	rs1151996	12420807	0.36	A/C	12420807	intron
	145	rs1151997	12421004	0.36	A/G	12421004	intron
	146	rs1151999	12422153	0.36	G/T	12422153	intron
	147	rs4135280	12423994	0.244	C/T	12423994	intron
	148	rs796313	12424528	0.349	G/T	12424528	intron
	149	rs796290	12424683	0.372	C/G	12424683	intron
	150	rs4135283	12424793	0.244	A/C	12424793	intron
	151	rs1822825	12424963	0.36	A/G	12424963	intron
	152	rs709149	12425354	0.36	A/G	12425354	intron
	153	rs709150	12426337	0.36	C/G	12426337	intron
	154	rs709151	12429999	0.36	C/T	12429999	intron
	155	rs709154	12431834	0.375	A/T	12431834	intron
	156	rs709156	12436615	0.128	A/G	12436615	intron
	157	rs1175540	12440243	0.372	A/C	12440243	intron
	158	rs1175542	12441214	0.372	A/G	12441214	intron
	159	rs1175543	12441433	0.384	A/G	12441433	intron
	160	rs1177809	12441490	0.349	A/G	12441490	intron
	161	rs1175544	12442044	0.349	C/T	12442044	intron
	162	rs1797912	12445239	0.384	A/C	12445239	intron
	163	rs3856806	12450557	0.244	C/T	12450557	synonymous codon
	164	rs1152003	12452055	0.477	C/G	12452055	3′flanking

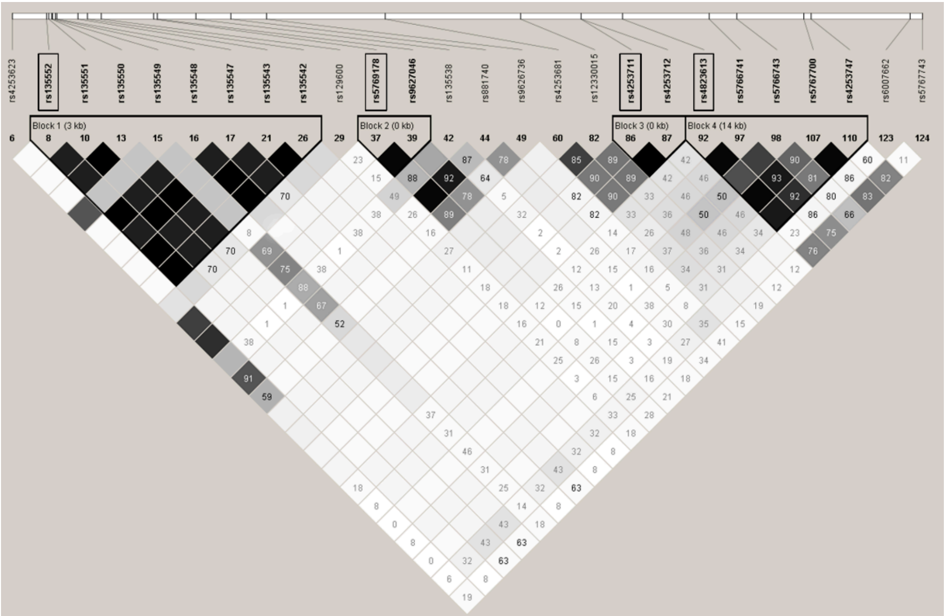

TagSNP	r^2-value	alleles	MAF
rs135551		A/G	0. 081
rs135550	1		
rs135549	0. 231		
rs135548	0. 846		
rs135547	1		
rs135543	0. 846		
rs135542	0. 846		
rs135552	0. 846		
rs5769178		A/C	0. 221
rs9627046	0. 933		
rs4253711		A/G	0. 156
rs4253712	1		
rs4823613		A/G	0. 221
rs5766741	0. 933		
rs5766743	0. 686		
rs5767700	0. 933		
rs4253747	0. 869		

Figure S1. Linkage disequilibrium (LD) plot of the SNPs with a MAF ⩾ 5% within the *PPARα* gene and 3 kb up- and down-stream regions are displayed using an r^2 black and white color scheme. Black represents very high LD ($r^2 = 1$), and white indicates the absence of correlation ($r^2 = 0$) between SNPs. The selected SNPs and SNPs that are indirectly measured by them are listed with corresponding r^2 values. The alleles of selected SNPs are given with their frequencies, according to the HapMap data for Chinese individuals from Beijing.

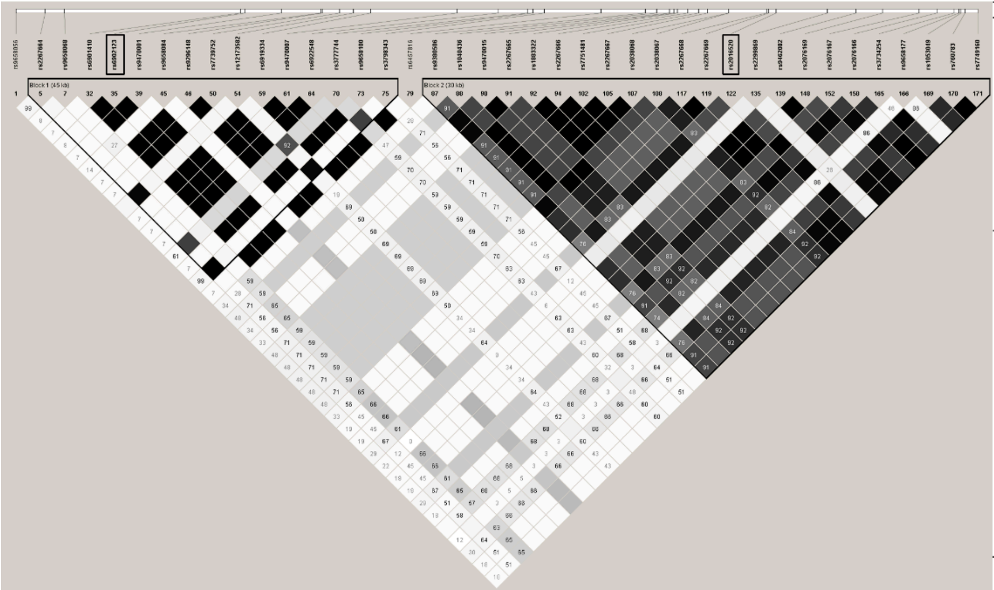

TagSNP	r^2-value	alleles	MAF
rs6902123		C/T	0. 056
rs9470001	1		
rs9658084	0. 004		
rs9296148	1		
rs7739752	1		
rs12173582	0. 02		
rs6919334	1		
rs9470007	1		
rs6922548	1		
rs3777744	0. 151		
rs9658100	1		
rs3798343	0. 019		
rs2267664	0. 018		
rs9658068	0. 006		
rs6901410	1		
rs2016520		C/T	0. 233
rs1040436	0. 882		
rs1053049	0. 936		
rs1883322	0. 882		
rs2038067	0. 571		
rs2038068	0. 882		
rs2076166	0. 754		
rs2076167	1		
rs2076169	0. 697		
rs2267665	0. 571		
rs2267666	0. 882		
rs2267667	0. 882		
rs2267668	0. 697		
rs2267669	0. 697		
rs2299869	0. 069		
rs3734254	0. 93		
rs760783	0. 754		
rs7749160	0. 754		
rs7751481	0. 882		
rs9380506	0. 502		
rs9462082	0. 697		
rs9470015	0. 571		
rs9658177	0. 029		

Figure S2. Linkage disequilibrium (LD) plot of the SNPs with a MAF ≥ 5% within the *PPAR*β gene and 3 kb up- and down-stream regions are displayed using an r^2 black and white color scheme. Black represents very high LD ($r^2 = 1$), and white indicates the absence of correlation ($r^2 = 0$) between SNPs. The selected SNPs and SNPs that are indirectly measured by them are listed with corresponding r^2 values. The alleles of selected SNPs are given with their frequencies, according to the HapMap data for Chinese individuals from Beijing.

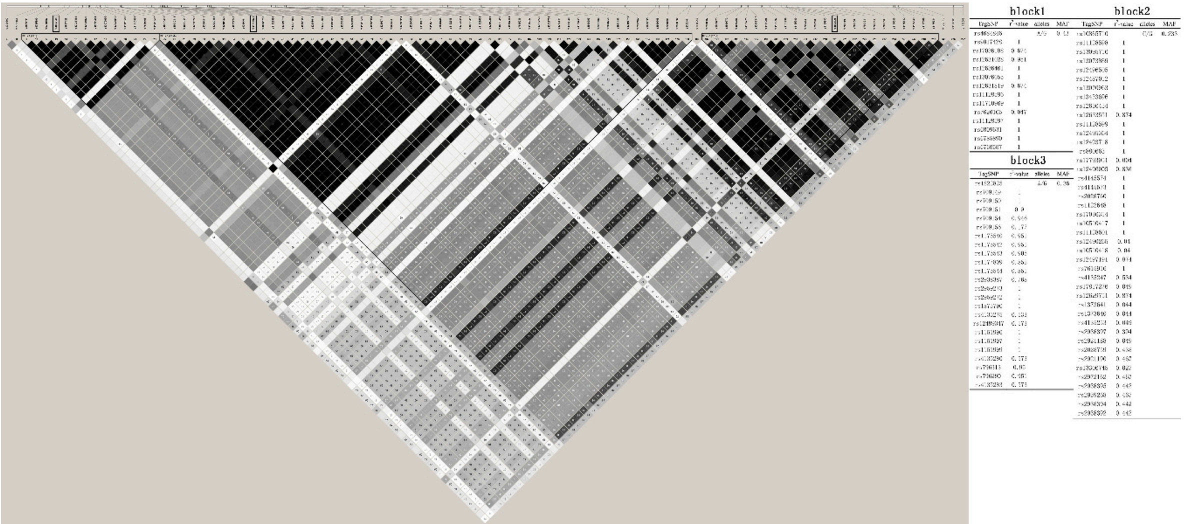

Figure S3. Linkage disequilibrium (LD) plot of the SNPs with a MAF ≥ 5% within the *PPAR*γ gene and 3 kb up- and down-stream regions are displayed using an r^2 black and white color scheme. Black represents very high LD ($r^2 = 1$), and white indicates the absence of correlation ($r^2 = 0$) between SNPs. The selected SNPs and SNPs that are indirectly measured by them are listed with corresponding r^2 values. The alleles of selected SNPs are given with their frequencies, according to the HapMap data for Chinese individuals from Beijing.

© 2016 by the authors; licensee MDPI, Basel, Switzerland. This article is an open access article distributed under the terms and conditions of the Creative Commons by Attribution (CC-BY) license (http://creativecommons.org/licenses/by/4.0/).
